# Prevalence of Venous Thromboembolism in Critically Ill Patients With Coronavirus Disease 2019: A Meta-Analysis

**DOI:** 10.3389/fmed.2021.603558

**Published:** 2021-04-29

**Authors:** Changgang Wu, Yunlong Liu, Xiangjing Cai, Wenming Zhang, Yongjie Li, Chunsheng Fu

**Affiliations:** ^1^Department of Respiration, Liaocheng Infectious Disease Hospital, Liaochen, China; ^2^Department of Infectious Diseases, Liaocheng Infectious Disease Hospital, Liaocheng, China; ^3^Department of Critical Care Medicine, Liaocheng Infectious Disease Hospital, Liaocheng, China

**Keywords:** coronavirus disease 2019, critically ill, venous thromboembolism, prevalence, meta-analysis

## Abstract

**Background:** Accumulating evidence suggests that coronavirus disease 2019 (COVID-19) is associated with hypercoagulative status, particularly for critically ill patients in the intensive care unit. However, the prevalence of venous thromboembolism (VTE) in these patients under routine prophylactic anticoagulation remains unknown. A meta-analysis was performed to evaluate the prevalence of VTE in these patients by pooling the results of these observational studies.

**Methods:** Observational studies that reported the prevalence of VTE in critically ill patients with COVID-19 were identified by searching the PubMed and Embase databases. A random-effect model was used to pool the results by incorporating the potential heterogeneity.

**Results:** A total of 19 studies with 1,599 patients were included. The pooled results revealed that the prevalence of VTE, deep venous thrombosis (DVT), and pulmonary embolism (PE) in critically ill patients with COVID-19 was 28.4% [95% confidence interval (CI): 20.0–36.8%], 25.6% (95% CI: 17.8–33.4%), and 16.4% (95% CI: 10.1–22.7%), respectively. Limited to studies, in which all patients received routine prophylactic anticoagulation, and the prevalence for VTE, DVT, and PE was 30.1% (95% CI: 19.4–40.8%), 27.2% (95% CI: 16.5–37.9%), and 18.3% (95% CI: 9.8%−26.7%), respectively. The prevalence of DVT was higher in studies with routine screening for all patients, when compared to studies with screening only in clinically suspected patients (47.5% vs. 15.1%, *P* < 0.001).

**Conclusion:** Critically ill patients with COVID-19 have a high prevalence of VTE, despite the use of present routine prophylactic anticoagulation.

## Introduction

The novel coronavirus (SARS-CoV-2)-infected disease (COVID-19) occurred in Wuhan in December 2019. Since then, the worldwide spread of the disease began (1, 2). The disease was characterized by respiratory and systemic illness, and approximately 10–15% of patients have been reported to progress to severe pneumonia (3, 4). These critically ill patients with COVID-19 often have acute respiratory distress syndrome and multiple-organ failure, which needs to be treated in the intensive care unit (ICU) (1). In addition, accumulating evidence from clinical observations suggests that patients with COVID-19 may be associated with hypercoagulative status, particularly for those who are critically ill, which may lead to a high incidence of venous thromboembolism (VTE) for these patients (5–9). Two autopsy studies in deceased patients with COVID-19 revealed a very high prevalence of deep vein thrombosis (DVT, 58%) and pulmonary embolism (PE, 73%) (10, 11). However, VTE was not even clinically suspected antemortem in any of these patients (10, 11). Since the incidence of VTE has been well-recognized as an independent risk factor for mortality in critically ill patients, including sepsis and septic shock (12–14), it is important to estimate the prevalence of VTE in critically ill patients with COVID-19, particularly for those who received prophylactic anticoagulation under the present guidelines. Emerging data regarding the prevalence of VTE in critical ill patients with COVID-19 have been reported in recent observational studies (15–33), and the results varied. The present study aimed to evaluate the prevalence of VTE in these patients by pooling the results of these observational studies.

## Methods

The systematic review and meta-analysis was designed and performed in accordance with the Meta-analysis of Observational Studies in Epidemiology (MOOSE) (34) and Cochrane's Handbook (35) guidelines.

### Literature Searching

The PubMed and Embase databases were systematically searched using a search strategy of combined terms. The combined terms were entered into PubMed as a single search, as (“coronavirus” OR “severe acute respiratory syndrome coronavirus 2” OR “SARS-CoV-2” OR “novel coronavirus” OR “nCoV” OR “2019-nCoV” OR “COVID-19”) AND (“pulmonary embolism” OR PE OR “deep venous thrombosis” OR DVT OR “venous thromboembolism” OR VTE OR thrombosis OR embolism OR thrombus OR thrombli OR embolization OR thromboembolism). We used this keyword search strategy instead of those searched as “text words” or as “Mesh terms” or “Emtree” to retrieve more comprehensive records. The Cochrane's Library database was not searched because this study was a meta-analysis of observational studies rather than clinical trials (randomized controlled trials). The search was limited to human studies published in the English or Chinese language. The reference lists of the original and review articles were also manually screened. The final literature search was performed on July 4, 2020.

### Inclusion and Exclusion Criteria

Studies that fulfilled the following criteria were included: (1) published as full-length articles in the English or Chinese language in peer-reviewed journals; (2) designed as observational studies that include critically ill patients with COVID-19, who were treated in the ICU, or a subgroup data of critically ill patients was reported; (3) included adult patients (≥18 years of age) that were screened for the events of VTE, DVT, or PE, with imaging studies such as duplex ultrasound scanning (DUS) and/or computed tomography pulmonary angiogram (CTPA); and (4) reported the prevalence of VTE, DVT, or PE during the ICU stay. If studies with overlapping participants were encountered, reports with a larger sample size were included. Abstracts, reviews, preclinical studies, and studies published in preprints that were not peer-reviewed were excluded.

### Data Extraction and Quality Evaluation

The literature search, data extraction, and quality assessment were independently performed by two authors according to the predefined inclusion criteria. Discrepancies were resolved by consensus. Data on the characteristics of these studies were extracted: name of the first author, year of publication, country where the study was conducted, sample size, mean age, proportion of males, number of patients with chronic lung disease, prevalence of diabetes, status for prophylactic anticoagulation before screening, strategy for VTE diagnosis, and number of patients with each VTE outcome. The modified Newcastle–Ottawa Scale (NOS) (36) was used to evaluate the quality of the included studies, which predominantly focused on the aspects of the selection of the study groups and the ascertainment of the outcome of interest. Moreover, we used the Grading of Recommendations Assessment, Development and Evaluation (GRADE) approach to assess the quality of the body of evidence (37). The GRADE methodology involves rating the initial quality of observational data as “low,” followed by upgrading based on three criteria (large effect size, dose-response gradient, and plausible confounding) (38).

### Statistical Analyse

The data of prevalence and its corresponding stand errors (SEs) were calculated from the 95% CIs or *P-*values and were logarithmically transformed to stabilize variance and normalize the distribution. For studies that did not report the prevalence data, data regarding the incident cases of VTE outcomes and overall enrolled patients were extracted. The Cochrane's Q test and I^2^ test were used to evaluate the heterogeneity among the include cohort studies (39). A significant heterogeneity was considered when I^2^ >50%. A random-effect model was used to pool the results, since the model was considered to incorporate the potential heterogeneity and could therefore derive a more generalized result (35). Furthermore, a subgroup analysis was also performed to evaluate the potential influence of prophylactic status and VTE screening strategy on the outcome. The STATA software (Version 12.0; Stata Corporation, College Station, TX, USA) was used for the statistical analyses.

## Results

### Literature Search Results

**Figure 1:**
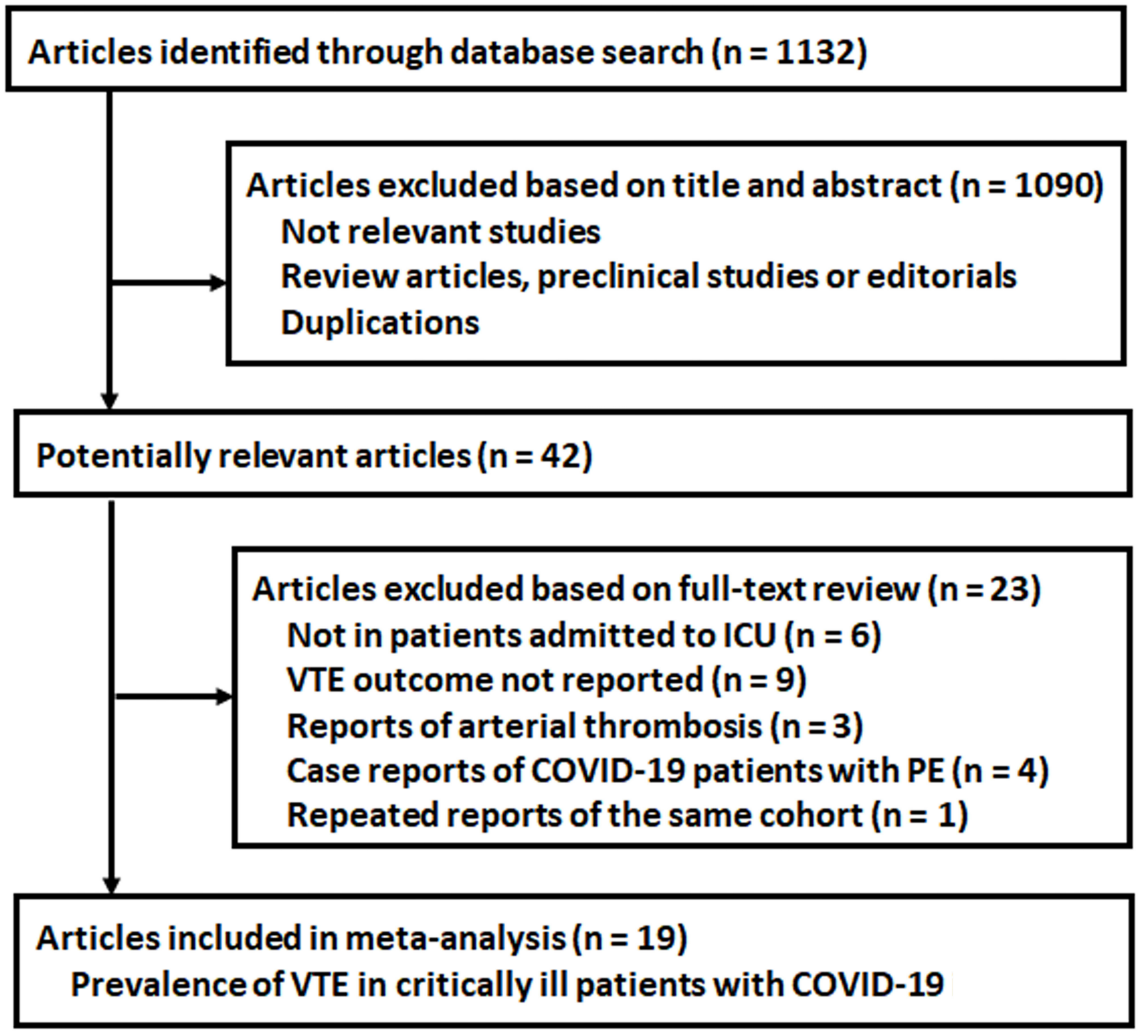
Flowchart for the literature search.

The process for the literature search and study identification is summarized in Figure 1. Briefly, 1,132 records were identified after the initial database search and after excluding duplicated records. The further screening with the titles and abstracts further excluded 1,090 records, and this is mainly because these were irrelevant to the aim of the study. For the 42 records that underwent full-text review, 23 studies were further excluded due to the reasons listed in Figure 1. Overall, 19 observational studies met the inclusion criteria of the meta-analysis.

### Study Characteristics and Quality Evaluation

A total of 19 studies with 1,599 critically ill patients with COVID-19 were included (15–33). The characteristics of these included studies are summarized in Table 1. These studies were performed in China, the United States, the United Kingdom, France, Italy, the Netherlands, and Spain. All of these were retrospective, except for four studies, which were prospective (23–25, 28). The number of critically ill patients with COVID-19 included in each study varied within 24–184. The mean age of patients varied within 53–68 years old, with the proportion of male patients ranging within 46–81%. All patients received anticoagulation before screening for VTE in 14 studies (16, 18, 20–22, 24, 26–33), while a part of the patients received anticoagulation before screening in the other five studies (15, 17, 19, 23, 25). In all of the included studies, the PE events were screened by CTPA only in clinically suspected patients. As for DVT, all patients were routinely screened with DUS in five studies (15, 24–26, 28), while in the other nine studies, only clinically suspected DVT patients were screened (17, 18, 20, 23, 29–33). The details of the quality evaluation according to the modified NOS are shown in Table 2. The scales for the included studies varied within 3–5. Following the GRADE methodology, we graded the quality of evidence for the outcome “prevalence of VTE events” to be low because risk of bias of inconsistency and indirectness may exist (Table 3).

**Table 1:** Characteristics of the included studies.

**Study**	**Country**	**Design**	**Sample size**	**Mean age**	**Male**	**Chronic lung disease**	**DM**	**Use of anticoagulation before screening**	**Strategy for VTE diagnosis**	**No. of VTE Pts**	**No. of DVT Pts**	**No. of PE Pts**
				**years**	**%**	**%**	**%**	**%**				
Hippensteel et al. (15)	USA, Colorado	RC	91	56.5	58.2	18.7	30.8	Partial	Screen for clinically suspected VTE, routine screen for DVT with DUS, and suspected PE with CTPA	24	19	5
Fraisse et al. (18)	France, Argenteuil	RC	92	61	79	20	38	All	Screen for clinically suspected VTE	31	12	25
Hekimian et al. (19)	France, Paris	RC	51	NR	NR	NR	NR	Partial	Screen for clinically suspected PE with CTPA	NR	NR	8
Maatman et al. (20)	USA, Indianapolis	RC	109	61	57	31	39	All	Screen for clinically suspected VTE, DUS for DVT, and CTPA for PE	31	30	5
Poissy et al. (16)	France, Lille	RC	107	57	59.1	NR	NR	All	Screen for clinically suspected PE with CTPA	NR	NR	22
Zhang et al. (17)	China, Wuhan	RC	143	63	57.1	NR	18.2	Partial	Screen for clinically suspected VTE, DUS for DVT	NR	66	NR
Criel et al. (22)	Belgium, Genk	RC	30	64.5	67	NR	17	All	Screen for clinically suspected VTE	4	NR	NR
Bompard et al. (21)	France, Paris	RC	24	64	70	NR	NR	All	Screen for clinically suspected PE with CTPA	NR	NR	12
Helms et al. (23)	France, multicenter	PC	150	53	81.3	14	20	Partial	Screen for clinically suspected VTE, DUS for DVT, and CTPA for PE	27	3	25
Voicu et al. (24)	France, Paris	PC	56	NR	75	NR	45	All	Routine screen for DVT with DUS	NR	26	NR
Nahum et al. (28)	France, Saint-Denis	PC	34	62.2	78	6	44	All	Routine screen for DVT with DUS	NR	27	NR
Cui et al. (25)	China, Wuhan	RC	81	59.9	46	NR	10	Partial	Routine screen for DVT with DUS	NR	20	NR
Llitjos et al. (26)	France, Paris	RC	26	68	77	NR	NR	All	Routine screen for DVT with DUS	18	18	6
Middeldorp et al. (27)	Netherlands, Amsterdam	RC	75	61	66	NR	NR	All	Screen for clinically suspected VTE, DUS for DVT, and CTPA for PE	35	NR	NR
Desborough et al. (30)	UK, London	RC	66	59	73	9	41	All	Screen for clinically suspected VTE, DUS for DVT, and CTPA for PE	11	10	5
Klok et al. (31)	Netherlands, multicenter	RC	184	64	76	NR	NR	All	Screen for clinically suspected VTE, DUS for DVT, and CTPA for PE	68	3	65
Lodigiani et al. (32)	Italy, Milan	RC	61	61	80.3	NR	18	All	Screen for clinically suspected VTE, DUS for DVT, and CTPA for PE	4	2	2
Demelo et al. (29)	Spain, Madrid	PC	156	68.1	65.4	NR	NR	All	Screen for clinically suspected DVT with DUS	NR	23	NR
Thomas et al. (33)	UK, Cambridge	RC	63	NR	69	NR	NR	All	Screen for clinically suspected VTE, DUS for DVT, and CTPA for PE	17	12	5

*DM, diabetes mellitus; VTE, venous thromboembolism; DVT, deep venous thrombosis; PE, pulmonary embolism; RC, retrospective cohort; PC, prospective cohort; NR, not reported; DUS, duplex ultrasound scanning; CTPA, computed tomography pulmonary angiogram; Pts, patients*.

**Table 2:** Details of the study quality evaluation.

**Study**	**Representativeness of the cohort**	**Confirmed diagnosis of COVID-19**	**Reporting study protocol and all pre-specified outcomes**	**Validated assessment of outcome**	**Other bias**	**Overall quality**
Hippensteel et al. (15)	1	1	1	1	1	5
Fraisse et al. (18)	1	1	0	0	1	3
Hekimian et al. (19)	0	1	0	1	1	3
Maatman et al. (20)	1	1	1	1	1	5
Poissy et al. (16)	1	1	0	1	1	4
Zhang et al. (17)	1	1	1	1	1	5
Criel et al. (22)	1	1	0	0	1	3
Bompard et al. (21)	1	1	0	0	1	3
Helms et al. (23)	1	1	1	1	1	5
Voicu et al. (24)	1	1	0	1	1	4
Nahum et al. (28)	0	1	0	1	1	3
Cui et al. (25)	1	1	0	1	1	4
Llitjos et al. (26)	1	1	0	0	1	3
Middeldorp et al. (27)	1	1	1	1	1	5
Desborough et al. (30)	1	1	1	1	1	5
Klok et al. (31)	1	1	1	1	1	5
Lodigiani et al. (32)	1	1	1	1	1	5
Demelo et al. (29)	1	1	1	1	1	5
Thomas et al. (33)	1	1	1	1	1	5

*COVID-19, coronavirus disease 2019*.

**Table 3:** Summary of findings table.

**Prevalence of venous thromboembolism in critically ill patients with coronavirus disease 2019**			
**Patient or population: critically ill patients with confirmed coronavirus disease 2019**			
**Settings: intensive care unit**			
**Outcomes**	**Prevalence (95% CI)**	**No. of participants (studies)**	**Quality of the evidence (GRADE)**
Venous thromboembolism Follow-up: during ICU stay	28.4% (20.0–36.8%)	947 (11 studies)	⊕⊖⊖⊖ low^a^^,^ ^b^
Deep venous thrombosis	25.6%	1312	⊕⊖⊖⊖ low^a^^,^ ^b^
Follow-up: during ICU stay	(17.8–33.4%)	(14 studies)	
Pulmonary embolism	16.4%	1024	⊕⊖⊖⊖ low^a^^,^ ^b^
Follow-up: during ICU stay	(10.1–22.7%)	(12 studies)	

*CI, Confidence interval; GRADE Working Group grades of evidence High quality, Further research is very unlikely to change our confidence in the estimate of effect. Moderate quality: Further research is likely to have an important impact on our confidence in the estimate of effect and may change the estimate. Low quality: Further research is very likely to have an important impact on our confidence in the estimate of effect and is likely to change the estimate. Very low quality: We are very uncertain about the estimate.*

a *Inconsistency: A considerable heterogeneity was detected which could not be explained by the proportions of patients with prophylactic anticoagulation or screening strategy for VTE (routine screening or screening only in clinically suspected patients).*

b *Indirectness: The validity of VTE outcomes (including DVT or PE) was not consistently reported among the included studies*.

### Prevalence of VTE in Critically Ill Patients With COVID-19

**Figure 2:**
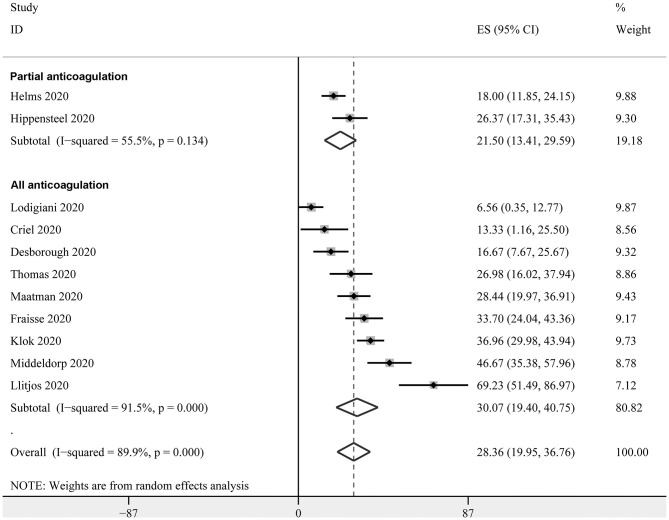
Forest plots for the meta-analysis of the prevalence of VTE in critically ill patients with COVID-19 stratified by the status of prophylaxis.

Eleven studies with 947 critically ill patients with COVID-19 (15, 18, 20, 22, 23, 26, 27, 30–33) reported the prevalence of VTE. Only clinically suspected patients were screened for VTE in these studies. The prevalence of VTE varied within 6.6%−69.3%, as reported in the individual studies. The pooled results with a random-effect model revealed that the overall prevalence of VTE in critically ill patients with COVID-19 was 28.4% (95% CI: 20.0–36.8%; Figure 2). The subgroup analysis revealed that the prevalence of VTE was 21.50% (95% CI: 13.4–29.6%) in studies with patients that partially received anticoagulation before screening, and 30.1% (95% CI: 19.4–40.8%) in studies with patients that all received anticoagulation. The different between subgroups was not significant (*P* = 0.31, Figure 2).

### Prevalence of DVT in Critically Ill Patients With COVID-19

**Figure 3:**
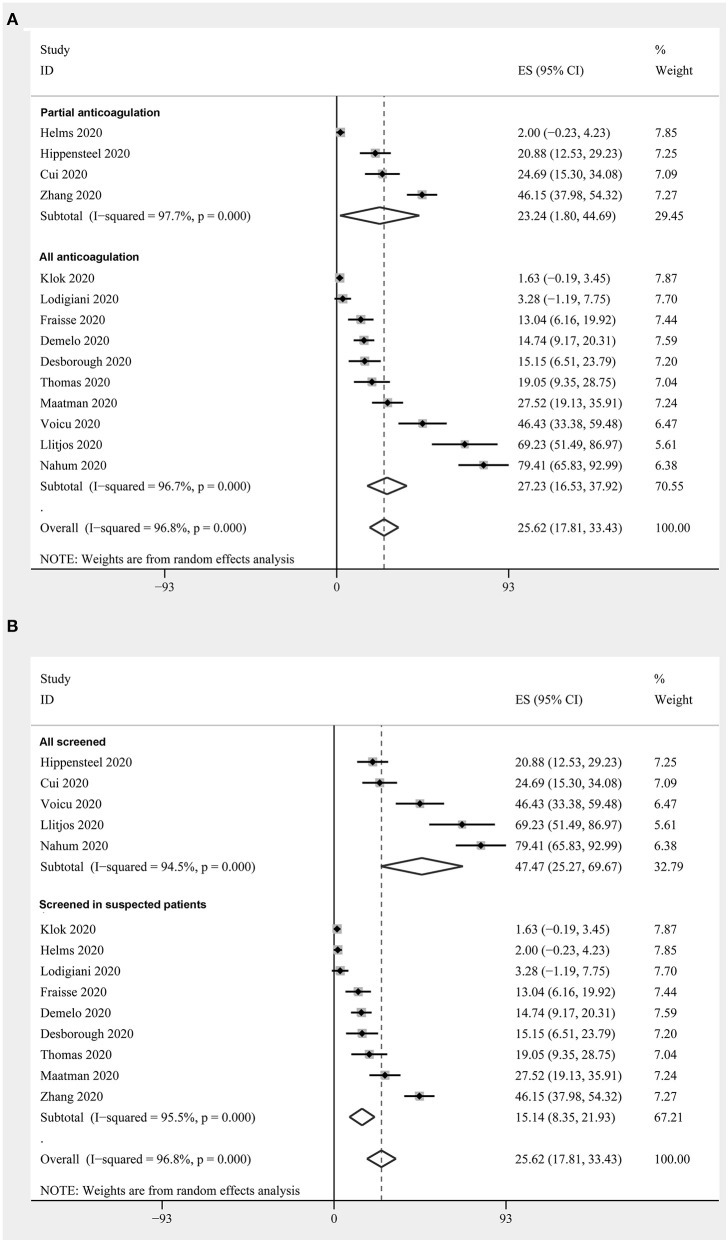
Forest plots for the meta-analysis of the prevalence of DVT in critically ill patients with COVID-19: **(A)** stratified by the status of the prophylaxis; **(B)** stratified by the screening strategy.

Fourteen studies with 1,312 critically ill patients with COVID-19 (15, 17, 18, 20, 23–26, 28–33) reported the prevalence of DVT. The pooled results revealed a prevalence of 25.6% (95% CI: 17.8–33.4%; Figure 3) in the overall patients. The subgroup analysis revealed a similar prevalence in studies with partial patients that received anticoagulation before screening (23.2%, 95% CI: 1.8–44.7%), and in studies where all patients received anticoagulation (27.2%, 95% CI: 16.5–37.9%) (*P* for subgroup difference = 0.94, Figure 3A). A significantly higher prevalence of DVT was observed in studies with routine screening for all patients (47.5%, 95% CI: 25.3–69.7%), when compared to studies with screening for only clinically suspected patients (15.1%, 95% CI: 8.4–21.9%) (*P* for subgroup difference < 0.001, Figure 3B).

### Prevalence of PE in Critically Ill Patients With COVID-19

**Figure 4:**
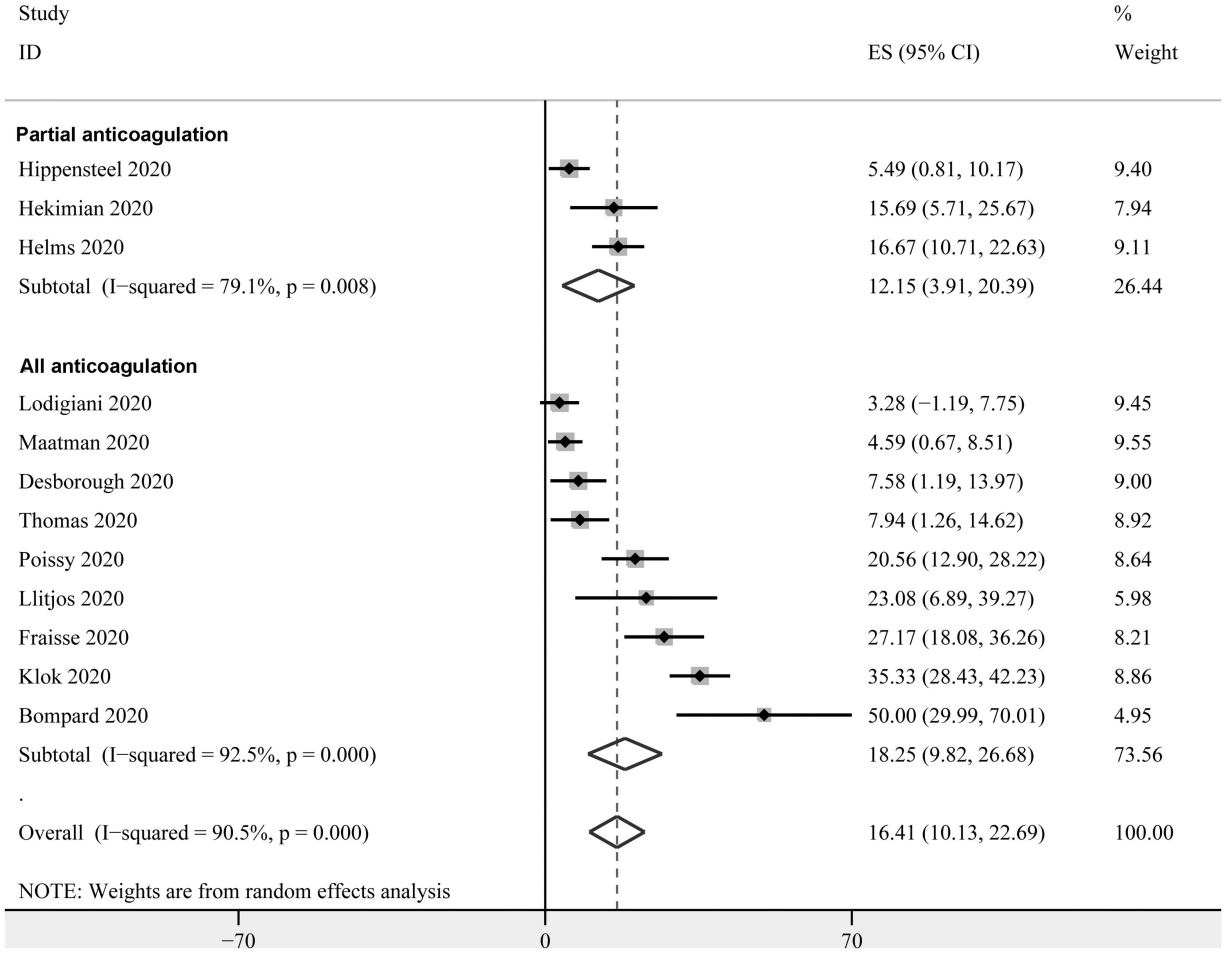
Forest plots for the meta-analysis of the prevalence of PE in critically ill patients with COVID-19 stratified by the status of prophylaxis.

Twelve studies with 1,024 critically ill patients with COVID-19 (15, 16, 18–21, 23, 26, 30–33) reported the prevalence of PE. Only clinically suspected patients were screened for PE in these studies. The pooled results revealed a prevalence of 16.4% (95% CI: 10.1–22.7%, Figure 4) for the overall patients. The subgroup analysis revealed a similar prevalence in studies with partial patients that received anticoagulation before screening (12.2%, 95% CI: 3.9–20.4%), and in studies where all patients received anticoagulation (18.3%, 95% CI: 9.8–26.7%) (*P* for subgroup difference = 0.70, Figure 4).

## Discussion

In the present meta-analysis of observational studies, it was found that the prevalence of VTE, DVT, and PE in critically ill patients with COVID-19 was 28.4, 25.6, and 16.4%, respectively. Limited to studies, in which all patients received routine prophylactic anticoagulation, the prevalence for VTE, DVT and PE was 30.1, 27.2, and 18.3%, respectively. The prevalence of DVT was higher in studies with routine screening for all patients, when compared to studies with screening only in clinically suspected patients (47.5 vs. 15.1%). Taken together, these results demonstrate that critically ill patients with COVID-19 have a high prevalence of VTE, despite the use of the present routine prophylactic anticoagulation. These findings highlight the importance for the awareness of the high incidence of VTE for clinicians in managing critically ill patients with COVID-19. Furthermore, these results suggest that the present routine prophylaxis for VTE in critically ill patients with COVID-19 may be inadequate.

Results of the meta-analysis revealed that critically ill patients with COVID-19 have a high prevalence of VTE, despite the use of the present routine prophylactic anticoagulation. In a previous large-scale cross-sectional study that included 11,111 critically ill medical patients who received chemoprophylaxis, the overall prevalence of VTE was 6.5% (40), which is significantly lower than that in critically ill patients with COVID-19 found in the meta-analysis (28.4%). In a previous meta-analysis, critically ill patients were admitted in the ICU, and the mean prevalence of DVT was reported to be 12.7% (14), which was considerably lower than that for the critically ill patients with COVID-19 found in the present meta-analysis (25.6%). In addition, a recent study of critically ill patients in the ICU that received thromboprophylaxis also revealed a prevalence of PE of 4% (41), which was also significantly lower than that for the critically ill patients with COVID-19 in the present meta-analysis (16.4%). The results of the present meta-analysis revealed similar results, in terms of the prevalence of VTE to a previous prospective cohort study in patients with severe sepsis and septic shock (37.2%). During the preparation and peer review of the manuscript, some related meta-analyses and systematic reviews have been published regarding the prevalence of VTE in patients with COVID-19. By including 42 studies enrolling 8,271 patients, Malas et al. showed an overall VTE rate of 21% (95% CI:17–26%) in patients with COVID-19, and the pooled odds of mortality were 74% higher among patients who developed VTE compared to those who did not (42). A later meta-analysis showed that while critically ill COVID-19 patients are more likely to require corticosteroid treatment, it may be associated with increased risk of VTE and poor clinical prognosis (43). In a recently published cohort study of relatively long-term follow-up (over 3 months), Demelo-Rodríguezb et al. showed that in patients with COVID-19 and VTE, mortality and major bleeding were high and almost a third of deaths were VTE-related (44). These findings, together with the results of our meta-analysis, showed the high prevalence and severe prognostic influence of VTE in patients with COVID-19, which highlights the importance of thrombotic risk assessment and VTE prevention in these patients (45).

Furthermore, it was found that the prevalence of VTE and its components was not significantly reduced in the meta-analysis limited to studies of all patients receiving prophylactic anticoagulation. Similar to studies on other critically ill patients, the prevalence of VTE has been confirmed as an independent predictor of in-hospital mortality in critically ill patients with COVID-19 (15). Therefore, strategies for reducing the risk of VTE may improve the prognosis of these patients. A Chinese study of 449 patients with severe COVID-19 (22% on thromboprophylaxis) revealed that although no difference in 28-day mortality was detected in the overall patients with and without prophylaxis with heparin, the use of heparin was associated with improved survival in patients with sepsis-induced coagulopathy (46). Furthermore, in another multicenter study conducted in Spain, which included 2,075 hospitalized patients for COVID-19, it was shown that heparin use was associated with lower mortality in these patients after controlling for age, gender, saturation of oxygen, body temperature, and concurrent medications (47). Unfortunately, neither of these studies analyzed the incidence of VTE and major hemorrhagic events in these patients. Collectively, for critically ill patients with COVID-19 and without any contraindication, anticoagulant thromboprophylaxis should be recommended (48). Furthermore, since routine anticoagulant thromboprophylaxis appears not to be associated with reduced risk of VTE in these patients, it would be reasonable to determine whether intensive anticoagulant thromboprophylaxis could reduce the risk of VTE. However, it has to be mentioned that it is likely that critically ill patients with COVID-19 also have a higher risk of bleeding. Therefore, intensive anticoagulant thromboprophylaxis should not be recommended until clinical trials that systematically evaluate the influence of such prophylaxis on the risk of VTE, bleeding, and survival in critically ill patients with COVID-19 become available.

The mechanisms of the vulnerability of critically ill patients with COVID-19 to VTE events remain undetermined. Several possible mechanisms have been suggested according to previous clinical and preclinical studies, including cytokine storm with activation of leukocytes, endothelium, and platelets resulting in upregulation of tissue factor, activation of coagulation, thrombin generation and fibrin formation, and deranged coagulation with imbalances in plasminogen activator inhibitor-1, tissue factor pathway inhibitor, and activated protein C that promotes fibrin generation and limits fibrinolysis, hypoxic vaso-occlusion, and direct viral effects with cell activation (49–52). Future studies are needed to determine whether these are specific to SARS-CoV-2 infection or a final common pathway in the thromboinflammatory response to viral infections and a marker of disease severity.

The present study has limitations, which should be considered when interpreting the results. First, the sample size of the included studies was generally small, and most of the studies were retrospective. Accordingly, selection and recall biases may exist, which could confound the findings. Moreover, to the best of our knowledge, no consensus has reached for the validated tool for quality evaluation of studies with non-comparative longitudinal design. Therefore, the validity of the modified NOS applied in our study for non-comparative longitudinal studies remain to be determined. Second, heterogeneity was considerable among the included studies. Although the present subgroup analysis in DVT revealed that the screening strategy (all patients or only clinically suspected patients) could significantly affect the results, there were other factors that may also contribute to the heterogeneity, such as the disease status of patients, previous history of chronic lung disease, concurrent medications, and the use of venous catheters. The investigators were unable to evaluate the potential influences of these factors on the prevalence of VTE, because these data are rarely reported. In addition, detailed information of prophylaxis and diagnosis (including screening) of VTE are important factors that may affect the prevalence of VTE in critically ill patients with COVID-19. However, these data were also rarely reported in detail in the included studies. Third, the subgroup analysis for the influence of the prophylactic status of patients on the prevalence of VTE should be cautiously interpreted, because these results were based on the meta-analysis of data at the study level. Furthermore, the investigators could not directly compare the prevalence of VTE in patients with and without prophylactic coagulation due to lack of access to individual-patient data. Moreover, because of the everyday rapidly increased publications regarding COVID-19 due to the pandemic, it is impossible to include the newest studies in our meta-analysis. Accordingly, the data of the manuscript could only reflect the situation of early pandemic of COVID-19. Finally, the influences of drugs and doses of prophylactic coagulation on the prevalence of VTE in critically ill patients with COVID-19 could not be determined, since none of the included studies specifically reported the related data. For future studies, clinical trials are preferably warranted.

## Conclusions

In conclusion, the present meta-analysis demonstrated that critically ill patients with COVID-19 have a high prevalence of VTE, despite of use of the present routine prophylactic anticoagulation. Clinicians should always be aware of the high incidence of VTE in these patients, and the optimization of prophylactic coagulation for these patients may be needed to improve the prognosis.

## Data Availability Statement

The original contributions presented in the study are included in the article/supplementary material, further inquiries can be directed to the corresponding authors.

## Author Contributions

CW was responsible for the preparation of the manuscript. CW, YL, XC, and WZ were responsible for study selection and data collection. CW and YL developed the search strategy and conducted the search in consultation with CF. CF provided support for statistical analysis, conceived the project, and will guarantee the content of the review. All authors contributed to the article and approved the submitted version.

## Conflict of Interest

The authors declare that the research was conducted in the absence of any commercial or financial relationships that could be construed as a potential conflict of interest.

## Abbreviations

COVID-19: coronavirus disease 2019
VTE: venous thromboembolism
PE: pulmonary embolism
DVT: deep venous thrombosis
ICU: intensive care unit
DUS: duplex ultrasound scanning
CTPA: computed tomography pulmonary angiogram
NOS: Newcastle –Ottawa Scale.
